# Socioeconomic and Psychosocial Stressors Contributing to General Adult Community Mental Health Recovery Service Referrals in Epsom, Surrey, a Retrospective Case Note Analysis

**DOI:** 10.1192/bjo.2024.144

**Published:** 2024-08-01

**Authors:** Christine Fullerton, Afaf Qazi

**Affiliations:** 1 Surrey & Sussex Healthcare NHS Trust, Redhill, United Kingdom; 2 Surrey & Borders Partnership NHS Foundation Trust, Epsom, United Kingdom

## Abstract

**Aims:**

Referrals to secondary mental health services in the United Kingdom are at record levels. In the wake of the coronavirus pandemic and cost of living crisis, many experienced a deterioration in their social and financial circumstances. It is widely accepted that social determinants impact mental health and wellbeing. This analysis aimed to investigate socioeconomic and psychosocial stressors contributing to referrals to the Community Mental Health Recovery Service (CMHRS) for the general adult population in Epsom, Surrey.

**Methods:**

This retrospective case note analysis focused on Single Point of Access (SPA) referrals made to CMHRS Epsom between 1st September 2022 and 1st September 2023. A random number generator was used to select a cross-section of 30 cases from 141 referrals. Following exclusion criteria, 29 cases were examined using an ICD–10 social determinants of health (Z55-Z65) lens. Finally, thematic analysis was used to identify key socioeconomic and psychosocial factors impacting referred patients.

**Results:**

Patients were most commonly referred to CMHRS for presentations of suicidal ideation and self-harm (n = 13). Referrals were also related to symptoms of depression, anxiety and psychosis, the need for diagnostic clarity and for review of medication. All but one referral (n = 28) cited psychosocial stressors contributing to the patient’s presentation. Five key themes were identified. These were: current unemployment (n = 18), current housing and financial concerns (n = 18), ongoing social isolation (n = 19), relationship conflict and breakdown (n = 10) and a background of child sexual and physical abuse (n = 10). Protective factors, for those able to identify them, were exclusively linked to the patient’s social network (n = 22). Patients cited family members, friends, neighbours, the church and their pets as reasons to stay alive and accept support.

**Conclusion:**

This analysis concluded that referrals to secondary mental health services in Epsom are significantly associated with a person's current and historical social circumstances. Policies and services which provide early intervention support with housing, employment and finances are vital in reducing the mental distress of at-risk individuals while also reducing pressure on mental health services. Reinforcing community and social support systems may be key in helping patients buffer psychosocial stress. Further study on this issue, involving a larger cohort, would be beneficial.

